# Clinical Pathways in Knee and Hip Arthroplasty: Narrative Review on Sustainability, Quality, and Resource Management

**DOI:** 10.2196/78174

**Published:** 2025-10-14

**Authors:** Manuel Godinho, Filipe Maçães, Helena Gonçalves, Firmino Silva

**Affiliations:** 1 Departamento de Ortopedia e Traumatologia, Unidade Local de Saúde de Entre o Douro e Vouga, Rua. Dr. Cândido Pinho 5, Santa Maria da Feira, 4520-211, Portugal, 351 913297114; 2 Departamento de Ortopedia e Traumatologia, Unidade Local de Saúde de Gaia e Espinho, Vila Nova de Gaia, Portugal; 3 ISLA - Polytechnic Institute of Management and Technology, Research Center, Vila Nova de Gaia, Portugal; 4 COPELABS – Cognitive and People-centric Computing Research Unit, Lisbon, Portugal

**Keywords:** clinical pathways, knee arthroplasty, hip arthroplasty, length of stay, treatment duration, treatment cost, treatment efficiency

## Abstract

**Background:**

Increasing arthroplasty volumes are testing health care system capacity, budgets, and workforce resilience. Clinical pathways (CPWs) provide a practical, evidence-based structure that aligns perioperative actions from preparation through follow-up. In this review, we treat three aims as coprimary: quality (patient outcomes and adherence to best practice); resource management and efficiency at the episode level (eg, length of stay, perioperative flow, direct costs); and sustainability, defined as the ability to maintain high-quality services over time by optimizing financial, human, and environmental resources while safeguarding equitable access.

**Objective:**

This study aimed to describe the main CPW subtypes used in hip and knee arthroplasty and synthesize evidence on their effects on quality of care, resource management, and sustainability.

**Methods:**

We conducted a narrative review of studies indexed in PubMed and Cochrane (2013‐2024) that evaluated CPWs in total hip and knee arthroplasty. Screening and selection were documented with a PRISMA (Preferred Reporting Items for Systematic Reviews and Meta-Analyses)-style flow diagram for transparency, and findings were synthesized thematically.

**Results:**

Across CPW models, consistent signals of benefit were identified. Enhanced Recovery After Surgery (ERAS) pathways accelerate recovery and enable earlier discharge without increasing complications, often reducing opioid exposure and time to mobilization. Integrated Clinical Pathways improve standardization and multidisciplinary coordination across settings, reducing unwarranted variability and supporting safer transitions of care. Fast-track programs emphasize early mobilization and streamlined perioperative processes, improving patient flow and satisfaction while decreasing length of stay. Outpatient arthroplasty pathways allow same-day discharge in carefully selected, low-risk patients, reducing bed occupancy and freeing inpatient capacity. Virtual clinics support remote follow-up, patient education, and complication surveillance, decreasing unnecessary in-person visits and optimizing clinician time. Collectively, these pathways align quality improvement with sustainability by lowering bed-days, improving adherence to evidence-based practices, and enabling more efficient use of operating rooms, wards, and workforce.

**Conclusions:**

This review highlights the importance of CPWs in improving care delivery and patient outcomes in orthopedic surgery. Future efforts should refine CPWs, integrate digital tools and platforms, adopt standardized sustainability metrics, and stay flexible to evolving service demands.

## Introduction

### Background

Hip and knee arthroplasty volumes continue to rise worldwide, driven by population aging and lifestyle-related osteoarthritis. While these operations reliably restore function and relieve pain, their scale and cost intensity place sustained pressure on inpatient beds, operating theaters, budgets, and the clinical workforce, making the organization of care as critical as the technical act itself [1,2].

Clinical pathways (CPWs) provide an evidence-based, multidisciplinary structure that specifies what is done, when, and by whom across the perioperative continuum—from preparation and anesthesia and analgesia to mobilization, discharge readiness, and follow-up. By reducing unwarranted variation and embedding best practice locally, CPWs have been associated in arthroplasty with shorter length of stay, stable short-term safety, better adherence to recommended processes, more reliable transitions of care, and more predictable use of resources across settings [3-8].

In this context, we frame three coprimary aims for CPWs. Quality addresses patient outcomes and fidelity to evidence-based practice. Resource management and efficiency captures near-term, episode- or service-line performance (eg, length of stay, perioperative flow, discharge disposition, direct costs). Sustainability reflects the system’s ability to maintain high-quality services over time by optimizing financial, human, and environmental resources while safeguarding equitable access, conceptually distinct from, but complementary to, efficiency. As health systems adopt value-based models, CPWs offer a practical vehicle to advance all three aims within routine delivery, rather than trading one for another [9,10].

Patient-centered pathway variants further illustrate this alignment. Fast-track programs pair clear milestones and early mobilization with person-centered communication, supporting timely, safe discharge and high satisfaction; outpatient arthroplasty pathways extend this logic to same-day discharge in appropriately selected patients, relieving bed occupancy without an apparent safety penalty. Digital adjuncts—including protocolized virtual follow-up, structured remote review, and routine PROM capture, as well as patient infotainment systems for education—can standardize escalation criteria, triage unnecessary face-to-face visits, and help preserve in-person capacity for those who need it most [11-16].

### Prior Work

Beaupre et al [17] describe clinical pathways (CPWs) as frameworks that promote adherence to best practice and turn guidelines into coordinated local care. By making clear who does what, when, and for whom, CPWs reduce unwarranted variation between providers and help maintain consistent care quality across the perioperative journey—from preparation to recovery and follow-up [18].

To maintain clarity in terms in this review, we group the literature into five pathway models that are widely used in orthopaedics: Enhanced Recovery After Surgery (ERAS) [19,20] (evidence-based perioperative bundles to attenuate surgical stress and speed recovery); Integrated Clinical Pathways (ICPs) [21] (structured coordination across teams and care settings); Fast-track pathways [22] (early mobilisation, optimised multimodal analgesia, and clear daily milestones); Outpatient arthroplasty pathways [23] (same-day discharge for appropriately selected patients); and Virtual clinics [13] (protocol-driven remote follow-up and patient education). Although these labels sometimes overlap in the literature, we use them consistently as distinct operational models in this review; brief definitions appear in Table 1.

ERAS pathways prioritize multidisciplinary, evidence-based perioperative care to reduce surgical stress, shorten hospital stays, and accelerate functional recovery, particularly in elective arthroplasty cases. ICPs aim to coordinate care across settings and providers, improving consistency and optimizing resource use in complex surgeries requiring long-term follow-up. Fast-track pathways focus on early mobilization, minimally invasive techniques, and optimized pain management, facilitating safe and rapid discharge for low-risk patients. Outpatient Arthroplasty pathways support same-day discharge in selected patients, helping manage surgical backlogs and reduce costs while maintaining safety and satisfaction. Virtual clinics offer remote follow-up, patient education, and digital assessments, enhancing convenience and reducing unnecessary in-person visits. Each model contributes to the broader goal of delivering efficient, standardized, and patient-centered care in joint arthroplasty while addressing different facets of the surgical journey.

**Table 1.** Concise summary of each CPW subtype.

CPW subtype	Main features	Primary goals	Typical applications	Example benefits*
ERAS (Enhanced Recovery After Surgery) [19,20]	Multidisciplinary, evidence-based perioperative protocols to speed recovery	Reduce hospital stay, accelerate recovery	Elective hip and knee arthroplasty	Discharge in 0‐3 days, no increased complications [6,24]
ICP (Integrated Clinical Pathway) [21]	Structured care plans ensuring coordination across teams and settings	Improve consistency, reduce variability, optimize resources	Complex surgeries with extended follow-up	Reduced length of stay and costs, mixed results [7]
Fast-track Pathway [22[]	Protocols focusing on early mobilization and optimized pain control	Reduce hospital stay, reduce complications	Joint replacement, low-risk patients	Earlier discharge, fewer complications [22]
Outpatient Arthroplasty Pathway [23]	Protocols for same-day discharge in selected patients	Maximize efficiency, reduce costs	Elective arthroplasty, low-risk patients	85% same-day discharge, cost savings [12]
Virtual Clinic [13]	Digital platforms for remote follow-up and education	Improve convenience, reduce in-person visits	Post-operative follow-up in orthopedics	Fewer in-person visits, improved satisfaction [14]

### Objectives

This narrative review examines how CPWs in hip and knee arthroplasty affect three vectors: quality of care, resource management (efficiency), and sustainability.

*Quality* refers to clinical and patient-reported outcomes and adherence to evidence-based practices.

*Efficiency* denotes near-term, episode- or service-line performance (eg, length of stay, operating-room turnover, discharge readiness, episode cost).

**Figure 1.**
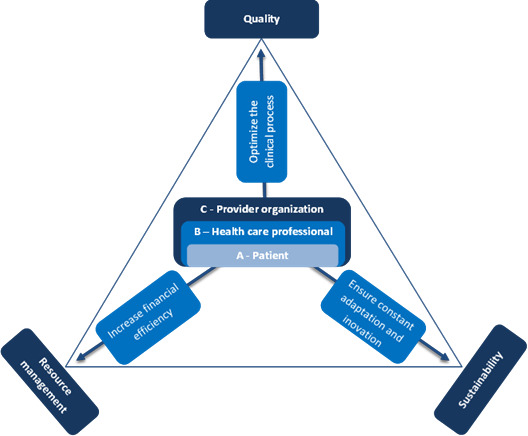
Three fundamental vectors in health care.

*Sustainability* is the health system’s capacity to maintain high-quality arthroplasty care over time while optimizing financial, human, and environmental resources and preserving equitable access. To avoid conflation, efficiency is treated as a short-term operational outcome, whereas sustainability captures durable, system-level impact across economic, workforce, environmental (proxy), and equity domains. Figure 1 illustrates the three-domain framework (quality, efficiency, sustainability) that guides this review.

Figure 1 Conceptual framework: clinical pathways optimize the clinical process at three stakeholder levels: patient, health care professional, and provider organization to align service quality, resource management (efficiency), and health-system sustainability.

## Methods

### Literature Search

**Figure 2.**
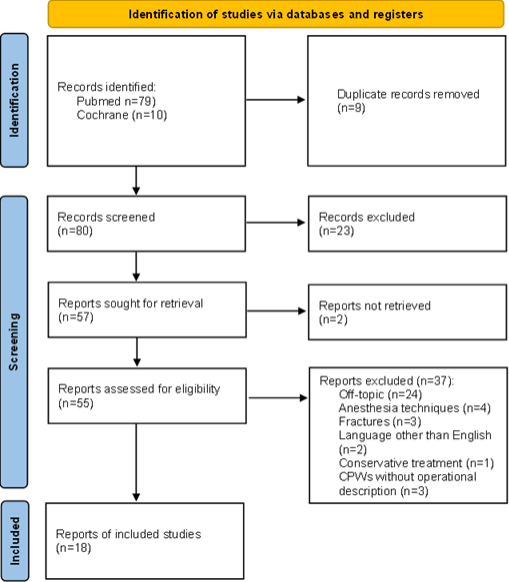
PRISMA flowchart diagram; CPWs - clinical pathways.

To explore the impact of CPWs in knee and hip arthroplasty, we conducted a literature search using PubMed and Cochrane databases, focusing on articles published in the past 12 years (January 2013 to December 2024). We used the MeSH terms “Critical Pathways”[Mesh] AND “Arthroplasty”[Mesh], including only English-language articles. While PRISMA [25] guidelines are typically used for systematic reviews, we adapted them to ensure a transparent and systematic literature search, given the diverse clinical pathways in joint arthroplasty. Studies were included if they addressed CPWs (including ERAS, ICPs, fast-track, outpatient arthroplasty pathways, or virtual clinics) for knee or hip arthroplasty, and reported outcomes related to quality, resource management or sustainability. Of the 89 articles identified, 18 met these criteria and were included in the review. The full study selection process and reasons for exclusion are detailed in Figure 2 (PRISMA flow diagram).

### Inclusion Criteria 

Studies of clinical pathways for hip arthroplasty, knee arthroplasty, or both, that reported outcomes related to sustainability, quality, or resource management were included.

### Exclusion Criteria 

Studies not addressing clinical pathways in hip or knee arthroplasty; articles not retrievable; articles not available in English were excluded.

### Data Extraction and Synthesis

Each included study was classified into one of five predefined pathway models (Table 1): ERAS, ICPs, fast-track programs, outpatient arthroplasty pathways, or virtual clinics. Mixed reports were assigned to the predominant subtype based on operational characteristics. Owing to heterogeneity in designs and outcomes, we extracted data as reported and synthesized findings narratively, organizing results under three co-primary aims: quality, resource management (efficiency), and sustainability (mapped to economic, operational, workforce, environmental-proxy, and equity or access domains).

### Ethical Considerations

This narrative review used only previously published data, with no new human subject involvement; therefore, Institutional Review Board review approval was not required.

## Results

### Overview of Clinical Pathway Subtypes

Across the 18 included studies, evidence addressed five predefined CPW models (Table 1): ERAS, ICPs, fast-track programs, outpatient arthroplasty pathways, and virtual clinics. Hybrid reports were assigned to the predominant subtype. Despite variation in scope and implementation, they share common goals of improving recovery, optimizing resource use, and standardizing care.

### Quality of Care

Applying standardized practices through clinical pathways reduces variability and increases consistency, both critical for successful elective joint replacement. This is underscored by the “halo effect” observed with care pathways, where improvements in one procedure translate to gains in others [26].

Within enhanced recovery programs, quality improvements are seen in adherence to recommended practices (for example, prophylactic antibiotics within 60 minutes and early postoperative physiotherapy), which support better processes and outcomes. Foni et al [5] report gains, including reduced length of stay and stronger adherence to best practices, changes that align with improved patient experience and satisfaction.

ICPs elevate postoperative quality by standardizing protocols across the continuum of care, which likely contributes to fewer complications and shorter hospital stays, with corresponding improvements in perceived care quality. Zhou et al [7] highlight favorable effects on quality and efficiency metrics; although direct satisfaction measures were not detailed, the outcome profile implies enhanced patient perceptions. Complementing this, preoperative physical therapy, often embedded within standardized pathways, has been associated with a marked reduction in use of post-acute services; Snow et al [27] documented a 29% decrease across skilled nursing facilities, home health, and inpatient rehabilitation, reinforcing a recovery model that promotes patient independence.

Fast-track programs maintain high-quality care through a person-centered approach from the decision for surgery through recovery. By providing consistent information and predictable milestones, these programs reduce anxiety and uncertainty and are associated with high patient satisfaction when communication and education are delivered effectively [11].

Outpatient arthroplasty pathways extend this patient-centered ethos with rigorous selection criteria, structured education on pain, mobility, and exercises, and robust follow-up. Peacock et al [12] show that such pathways sustain high-quality care while aligning with cost-reduction goals; patients in the pilot phase reported overall satisfaction with the care process.

Finally, virtual clinics and digital adjuncts strengthen follow-up quality. A standardized combination of patient-reported questionnaires and radiology reports helps identify patients who need in-person assessment, streamlining follow-up, reducing unnecessary visits, and improving satisfaction [14]. In parallel, patient infotainment systems have been associated with improved care processes—such as fewer medical orders while maintaining medical quality—likely by enhancing education, setting expectations, and promoting patient autonomy in care management [15].

### Resource Management

Clinical pathways in orthopedic surgery help reduce health care costs, primarily due to reduced complications and shorter hospital stays. Enhanced recovery protocols facilitate earlier discharges, which potentially decrease overall treatment costs and free inpatient capacity for other cases [6,28]. Additionally, a comparison study revealed that shortened hospital stays could lead to savings in subacute care, amounting to significant financial efficiencies [27]. Maintaining low readmission rates alongside shorter length of stay has further supported cost-efficiency, particularly in high-volume total joint arthroplasty (TJA) practices [22].

Within ICPs, economic findings have been mixed, several reports indicate substantial cost savings, while others highlight context-dependent variability and the need for further optimization [7]. Process improvements aligned with pathway implementation, such as Lean Six Sigma, have also been associated with more efficient bed turnover and reduced direct costs, reinforcing the operational value of standardized care processes [29].

Fast-track programs contribute to cost efficiency by promoting earlier discharge readiness and stable perioperative flow, helping services manage higher volumes without additional inpatient resource strain. In settings where low readmission rates are maintained, these programs reinforce the financial advantages of rapid recovery systems and minimize perioperative complications that drive costs [30].

The adoption of outpatient TJA pathways is increasingly recognized for its cost-effectiveness, producing substantial savings for both providers and patients through same-day discharge of carefully selected cases and reduced reliance on inpatient resources [12]. At the episode level, pathway implementation in total knee arthroplasty (TKA) has been associated with a mean cost decrease of about US $1252 per surgery, underscoring tangible financial benefits of standardized care bundles [8]. More broadly, the economic impact of defined care pathways includes reductions in hospital stay length and the use of post-acute services, with substantial cost differentials per episode [8].

Virtual clinics introduce additional savings by minimizing routine face-to-face follow-ups in hip and knee arthroplasty, optimizing the allocation of clinical resources and lowering the direct costs of outpatient services [13,14]. Beyond the index admission, preoperative physical therapy, often embedded within pathway frameworks, has been associated with decreased post-acute care use and related costs, further contributing to overall health care savings across the episode of care [27].

### Sustainability

Sustainability in arthroplasty relates to preserving system capacity and quality over time, economically, operationally, environmentally (by proxy), and in terms of equitable access, rather than only achieving short-term episode efficiency. Across CPW subtypes, sustainability signals were most evident, where earlier discharge and standardized transitions translated into fewer bed-days per 100 cases, steadier workflows, and avoidable follow-ups without a safety penalty.

Within ERAS, durable capacity gains derive from consistent reductions in bed-days at scale, with programs reporting earlier discharge and no increase in short-term complications or readmissions. These effects support predictable staffing, theater and ward use and are accompanied in several programs by lower episode costs, indicating economic sustainability beyond single-episode efficiency [6,8]. Environmentally, ERAS-driven bed-days avoided serve as a pragmatic proxy for reduced energy and water use at ward level, while standardized escalation criteria help preserve equity by ensuring timely access to higher-intensity care when needed.

ICPs contribute to sustainability by coordinating care across settings, reducing unwarranted variability and unplanned care and making workforce demand more predictable. Multisite implementations describe shorter length of stay (LOS) with maintained safety and generally favorable, though context-dependent, cost signals, consistent with economic and operational sustainability. Standardized transitions and bundle adherence also support continuity and equity, particularly when patient education and criteria for escalation are explicit [24].

In fast-track models, large cohorts demonstrate marked LOS reductions without increased readmissions, enabling services to expand activity while maintaining safety. The sustained drop in bed-days per 100 cases, coupled with earlier mobilization and discharge readiness, stabilizes perioperative flow and staffing needs, key features of workforce and operational sustainability. These effects also mitigate downstream use of post-acute services where pathways incorporate robust discharge planning and community coordination [22].

Outpatient arthroplasty pathways reconfigure care by shifting low-risk cases to same-day discharge under clear selection and escalation criteria. This preserves inpatient capacity for higher-acuity patients and, in successful implementations, lowers episode costs, aligning economic and operational sustainability with maintained access and patient experience (ie, equity). Where shorter admissions replace longer stays, comparative analyses suggest savings in subacute care, further reinforcing system-level benefits [8,12,23,31].

Finally, virtual clinics deliver a proportion of follow-ups remotely using protocolized questionnaires and standardized radiology reports, preserving access and triage while reducing in-person visits. This approach smoothens outpatient workloads (operational sustainability) and introduces an environmental proxy benefit via travel avoided, provided escalation criteria are well defined and safety signals remain acceptable. Programs report optimized clinic capacity and reduced direct outpatient costs, consistent with sustained service delivery at scale [7].

To consolidate the findings of our review, we mapped each CPW subtype against the three core domains explored, sustainability, quality, and resource management in Table 2. This comparative summary allows for a clearer understanding of how each model contributes across these dimensions.

**Table 2.** CPW subtypes by Sustainability, Quality, and Resource Management.

CPW subtype	Quality	Resource management	Sustainability
ERAS (Enhanced Recovery After Surgery)	High patient satisfaction and adherence to bundles; improved functional recovery in ERAS settings [24,28].	Earlier discharge and fewer bed-days per case; lower episode cost reported in pathway-based programs [8,11].	Discharge within 0‐3 days without increased morbidity/ morbidity/readmissions; updated ERAS protocols further reduce LOS [6,24] Accelerated functional recovery and shorter hospital stays [11] Reduced hospitalization times with updated ERAS protocols [6,28]
ICP (Integrated Clinical Pathway)	Reduces variability and ensures high-quality care in elective surgeries [7,27] Improves postoperative care and reduces complications [24] - Enhances adherence and satisfaction [7] Preoperative physical therapy reduces post-acute care use by 29% [27]	Subacute care savings from shorter stays [32]	Reduced hospital stay for lower limb surgeries [24] LOS reduction from 6.3 to 4.9 days, no increased readmissions [7] Standardization reduces recovery time and resource use [13]
Fast-track Pathway	High patient satisfaction with person-centered care [11,32]	Supports cost-efficiency in high-volume TJA practices [8]	Reduced stress and promoted early mobilization and recovery [22] Nurse-led care enhances efficiency and reduces per-patient resource use [12]
Outpatient Arthroplasty Pathway	High-quality care and strong patient satisfaction [12,23]	Post-acute care cost savings per episode [13]	84.6% same-day discharge success rate [23]
Virtual Clinic	Improves satisfaction via education and autonomy [22] Streamlined follow-up using questionnaires and radiology [5]	Mixed but promising cost reduction results [24]	- Reduced hospital stay for TKA [14] - Frees surgeon time by triaging unnecessary follow-ups [5]

This table compares five CPW subtypes—ERAS, ICP, Fast-track, Outpatient Arthroplasty, and Virtual Clinic—across three key dimensions: Sustainability, Quality, and Resource Management. ERAS promotes early discharge (0‐3 d), improves recovery and patient satisfaction, and reduces hospital and postoperative costs. ICP standardizes care to lower hospital stays and complications, enhances adherence to best practices, and provides cost savings with Lean process improvements. Fast-track supports early mobilization and nurse-led care, delivering high patient satisfaction while reducing readmissions and resource use. Outpatient Arthroplasty achieves high same-day discharge rates, ensures quality in low-risk surgeries, and significantly lowers financial burdens. Virtual Clinics enhance sustainability via remote follow-up, improve patient autonomy and satisfaction, and reduce outpatient and complication-related costs.

## Discussion

### Principal Findings

This review found that CPWs in hip and knee arthroplasty consistently improve near-term efficiency and quality while generating system-level sustainability gains, fewer bed-days per 100 cases, steadier perioperative workflows, and avoidable follow-up reductions. Benefits were observed across ERAS, ICPs, fast-track, outpatient pathways, and virtual clinics, with no apparent trade-off in short-term safety in appropriately selected cohorts.

### Indirect Outcomes

CPWs not only improve direct clinical outcomes but also lead to several indirect benefits. For example, the increased early mobilization and avoidance of continuous urinary catheters associated with CPWs have been linked to fewer complications and decreased readmission rates. Furthermore, this is underscored by the “halo effect” seen with care pathways, whereby improvements in one procedure translate to gains in others [26]. These indirect benefits highlight the comprehensive impact of CPWs on overall health care quality.

### Applicability of Clinical Pathways

The applicability of CPWs in orthopedics extends beyond immediate postoperative care. These pathways are crucial for addressing common problems encountered by patients, such as confusion about post-discharge medication and difficulty in assessing wound healing [31]. The integration of digital tools within CPWs can enhance patient education and engagement, providing clear guidance and support throughout the patient’s journey [30]. This approach not only improves patient satisfaction but also reduces the burden on health care providers.

### Importance of Initiating and Improving CPWs

Initiating and continuously improving CPWs is vital for maintaining and enhancing their effectiveness. The development of a standardized virtual clinic model for the follow-up of hip and knee arthroplasty patients, for instance, represents a significant innovation in orthopedic care delivery [14]. By leveraging expert consensus, this model aims to maintain the quality of care while reducing the resource burden of traditional follow-up methods, leading to enhanced patient satisfaction and cost efficiencies.

### Digital Potential in Clinical Pathways

The integration of digital tools in CPWs has the potential to revolutionize orthopedic care. Digital tools can improve synchronization across different health care settings, enhancing disease management and data analysis capabilities. This digital evolution supports informed decision-making and efficient resource management, resulting in cost savings for health care systems [27]. The use of patient infotainment systems, as demonstrated by Huang et al [15], can reduce hospital stays without compromising care quality, highlighting the potential of digital solutions in enhancing CPWs.

Future clinical pathways should be digitally integrated and informed by policy and incentive frameworks—such as the Health Information Technology for Economic and Clinical Health Act and the Centers for Medicare & Medicaid Services’ Promoting Interoperability program—with platform designs that support seamless integration with the electronic health record and efficient clinician review of patient-generated health data and patient-reported outcomes [16]. This adaptability will be critical to addressing rising care demands, workforce shortages, and maintaining high-quality, patient-centered outcomes [9]. 

Clinical pathways are key enablers of safe, efficient, and sustainable arthroplasty care. Standardized sustainability metrics and thoughtful integration of digital tools will be essential to maintain quality while expanding capacity for rising arthroplasty demand.

### Challenges and Future Directions

Despite the benefits, implementing CPWs also presents challenges. The tension between adhering to fast-track requirements and addressing individual patient needs highlights the complexities of maintaining quality while standardizing care [33]. Future efforts should focus on refining CPWs to balance standardization with personalized care, leveraging digital advancements to support these goals.

The rapid implementation of outpatient TJA pathways during the COVID-19 pandemic is an example of how CPWs can adapt to emerging challenges. This initiative by Peacock et al [12] demonstrated the feasibility of outpatient surgeries to alleviate the strain on inpatient resources while maintaining high standards of patient care and satisfaction. Such adaptive strategies provide valuable insights for surgical centers facing similar challenges, emphasizing the importance of flexibility and innovation in CPWs. 

The evolution of CPWs must align with new health care policies emphasizing value-based care and patient-reported outcomes (PROMs). In the United States, models like TEAM (Transforming Episode Accountability Model) and BPCI (Bundled Payments for Care Improvement) link reimbursement to PROMs (eg, HOOS Jr., KOOS Jr.), requiring CPWs to integrate these tools to remain compliant and improve care quality [2,34].

In Europe, initiatives like the Santeon Group in the Netherlands, Sweden’s Quality Registries, and Germany’s DiGA program demonstrate how digital and outcome-driven frameworks are being institutionalized [10,34,35]. The Horizon Europe agenda supports broader adoption through interoperability, PROM integration, and digital innovation [36].

A cross-national review by Srivastava et al [37] emphasizes key enablers for successful digital CPWs: coordinated governance, reimbursement models, workforce training, data sharing, and patient involvement. Countries such as Finland and the United Kingdom illustrate both progress and challenges, especially in IT integration and equitable access [38].

### Conclusion

This review underscores the essential role of clinical pathways in orthopedic surgery, particularly in knee and hip arthroplasties. Through standardizing patient care, optimizing resource use, and enhancing sustainability, CPWs significantly improve patient outcomes, reduce variability in clinical practice, and contribute to greater health care efficiency. The integration of digital tools, such as virtual clinics and patient engagement platforms, further amplifies these benefits by facilitating remote follow-up, improving patient satisfaction, and reducing unnecessary hospital visits. However, challenges persist in balancing standardized protocols with individualized patient care. Future research should focus on refining CPWs and aligning them with global health care policies.

## Supplementary material

10.2196/78174 Checklist 1 PRISMA 2020 checklist.

## Abbreviations

CPW: Clinical Pathway
ERAS: Enhanced Recovery After Surgery
ICPs: Integrated Clinical Pathways
LOS: length of stay
PRISMA: Preferred Reporting Items for Systematic Reviews and Meta-Analyses
PROM: Patient-Reported Outcome Measure
TJA: total joint arthroplasty
TKA: total knee arthroplasty
